# Neurite elongation is highly correlated with bulk forward translocation of microtubules

**DOI:** 10.1038/s41598-017-07402-6

**Published:** 2017-08-04

**Authors:** Ahmad I. M. Athamneh, Yingpei He, Phillip Lamoureux, Lucas Fix, Daniel M. Suter, Kyle E. Miller

**Affiliations:** 1 Department of Biological Sciences, Purdue University, West Lafayette, IN 47907 USA; 2 Purdue Institute for Integrative Neuroscience, Purdue University, West Lafayette, IN 47907 USA; 3 Bindley Bioscience Center, Purdue University, West Lafayette, IN 47907 USA; 4 Birck Nanotechnology Center, Purdue University, West Lafayette, IN 47907 USA; 5 Department of Integrative Biology, Michigan State University, East Lansing, MI 48824 USA

## Abstract

During the development of the nervous system and regeneration following injury, microtubules (MTs) are required for neurite elongation. Whether this elongation occurs primarily through tubulin assembly at the tip of the axon, the transport of individual MTs, or because MTs translocate forward in bulk is unclear. Using fluorescent speckle microscopy (FSM), differential interference contrast (DIC), and phase contrast microscopy, we tracked the movement of MTs, phase dense material, and docked mitochondria in chick sensory and *Aplysia* bag cell neurons growing rapidly on physiological substrates. In all cases, we find that MTs and other neuritic components move forward in bulk at a rate that on average matches the velocity of neurite elongation. To better understand whether and why MT assembly is required for bulk translocation, we disrupted it with nocodazole. We found this blocked the forward bulk advance of material along the neurite and was paired with a transient increase in axonal tension. This indicates that disruption of MT dynamics interferes with neurite outgrowth, not by disrupting the net assembly of MTs at the growth cone, but rather because it alters the balance of forces that power the bulk forward translocation of MTs.

## Introduction

The high personal and financial costs associated with neurological diseases and injury to the nervous system motivate the need for more effective therapies that promote neuronal regeneration^1^. Microtubules (MTs) are central to this process^2, 3^, yet the mechanisms of how MTs contribute to axonal elongation are not completely understood. Classic studies suggested that MTs do not move out of the neuronal cell body in bulk^4–6^. Paired with the observation that disruption of MT assembly at the growth cone blocked axonal elongation^7^, the logical interpretation of these findings was that MT assembly in the growth cone is responsible for axonal elongation. However, studies over the last two decades have demonstrated that short MTs are transported rapidly by MT-based motors through a stationary array of long MTs^8, 10^, individual MTs can slide rapidly in *Drosophila* ^9^, MTs translocate anterogradely in *Aplysia* growth cones in response to traction forces^11, 12^, and bulk forward advance of the cytoskeletal meshwork occurs during axonal elongation based on movements of beads bound to the axon, axonal branch points, and docked mitochondria^13, 14^. Somewhat remarkably, a detailed quantitative analysis of MT motion in the growth cone and distal axon along with a correlation to neurite outgrowth has never been conducted in freely growing neurons to this date. Whereas it is generally agreed that MT translocation is important for axonal elongation, whether it occurs primarily though bulk advance of the MTs^13^, Stop-and-Go transport of short MTs^15^, or MT sliding is unknown^9^. Furthermore, while it is clear that MT assembly is required for axonal elongation^2^, why even subtle disruption of MT dynamics^16–20^ slows neurite outgrowth remains unclear. Here, we conducted a quantitative analysis of MT movements in both vertebrate and invertebrate neurons to develop a better understanding of the relationships between MT translocation, MT assembly, and neurite outgrowth. We find that in both systems neurite elongation is highly correlated with bulk forward translocation of MTs. To better understand the role of MT assembly and dynamics in elongation, we added a low dose of nocodazole and monitored bulk translocation and neuronal tension. We found that tension increased and bulk transport was blocked when MT assembly was inhibited. This suggests disruption of MT dynamics interferes with neurite outgrowth because it alters the balance of forces that power the bulk forward translocation of MTs.

## Results

### Anterograde translocation of MTs and mitochondria in elongating axons

**Figure 1:**
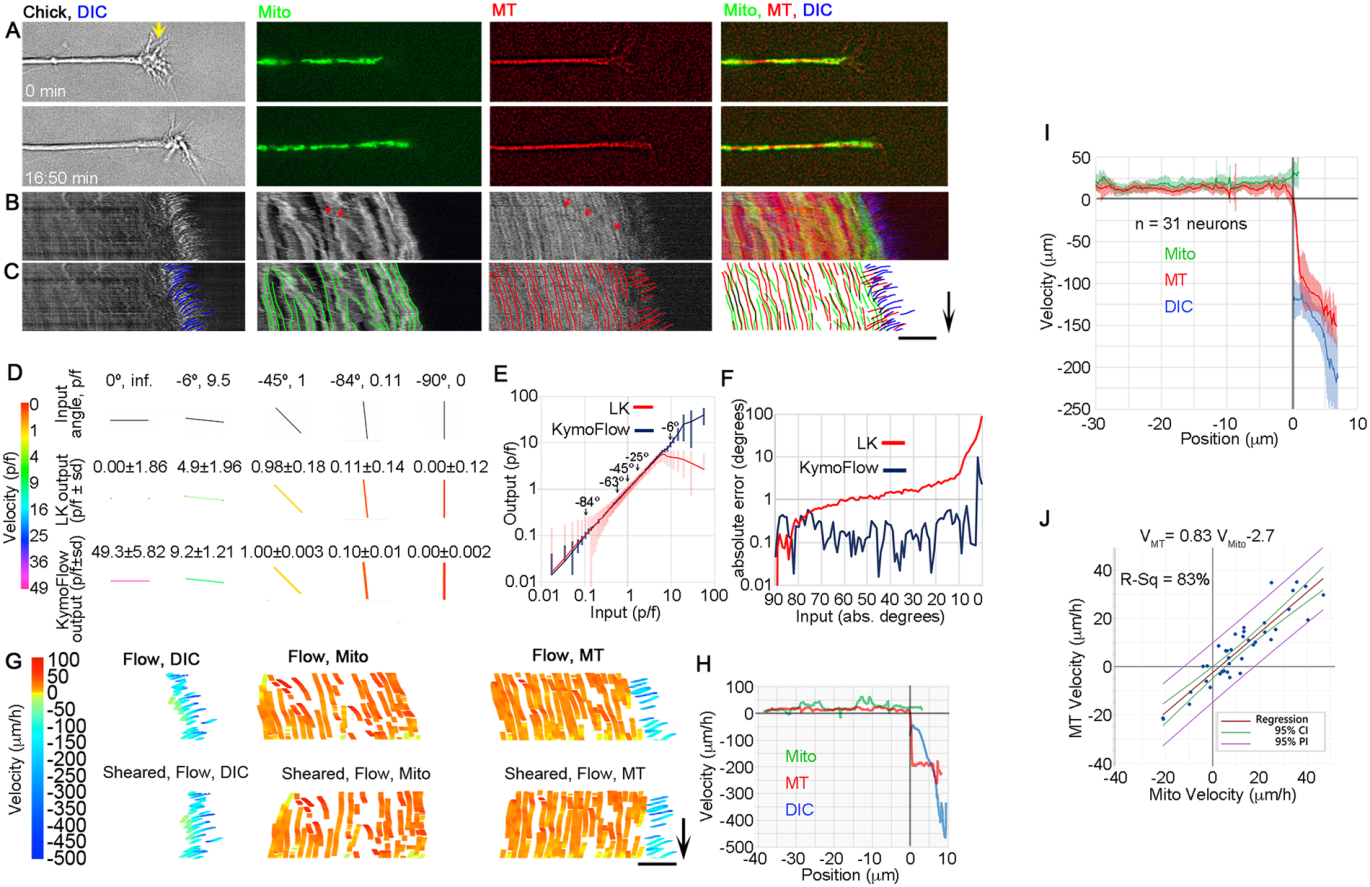
Axonal MTs and docked mitochondria translocate anterogradely during axonal elongation. (**A**) Still images of a chick DRG growth cone at the beginning and end of a time-lapse sequence in DIC, mitochondrial, MT fluorescent channels, and merged channels. Yellow arrow marks the T zone. (**B**) Kymograph showing motion. Red arrows indicate a mitochondrion and MT speckle undergoing fast transport. Scale and time bar as indicated. (**C**) Traces used to measure motion; arrow 10 min and bar 10 μm. (**D**) Input and output of LKMTA and KymoFlow algorithms for a subset of angles with color coded local velocities. (**E**) Input versus output shown as the average ± sd of velocity in units of p/f. (**F**) Average absolute error in degrees as a function of input angle. (**G**) Raw and sheared KymoFlow maps with color-coded velocities; arrow 10 min and bar 10 μm. (**H**) Velocity as a function of distance for the shown example. Position 0 corresponds with the growth cone T zone, where retrograde flow transitions into anterograde motion. (**I**) Velocity ± 95% CI as a function of distance for 31 neurons. (**J**) Regression analysis of docked mitochondrial and axonal MT velocity for 31 neurons.

Previous studies predominantly investigated rates of neurite elongation, MT translocation in neurites, or MT assembly in growth cones separately but did not attempt to correlate these critical parameters measured in the same cells. To directly observe the relationship between MT translocation, mitochondrial motion, and axonal elongation, chick sensory neurons were grown on laminin/poly-L-ornithine, microinjected with fluorescent tubulin, labeled with MitoTracker and imaged using DIC and fluorescent time lapse microscopy (Fig. 1A). To track motion, the movies (Video S1) were converted to kymographs (Fig. 1B), and lines were traced by hand using a drawing tablet over the paths of moving objects (Fig. 1C). Retrograde flow in the growth cone was analyzed using the DIC images, and the motion of docked mitochondria and slowly moving MT speckles were traced in both the growth cone and along the distal axon. As a working definition, fast moving objects were defined as those whose motion caused them to cross the paths of surrounding slower moving objects. Red arrows point out examples of such fast axonal transport of a mitochondrion and MT speckle moving at a rates of 0.07 μm/sec and 0.04 μm/sec respectively (Fig. 1B). Motion was analyzed in 34 chick sensory neurons over time spans of at least 15 minutes, but in some cases up to an hour for a total imaging time of 9:45 h. Because the rate of growth cone advance occasionally changed, some movies were divided into two or more parts, which yielded a total of 39 sequences. Of these, in 30 sequences neurites elongated at 24 ± 5 μm/h (mean ± 95% CI) and 9 retracted at −6 ± 5 μm/h (mean ± 95% CI). In the growth cone periphery, DIC refractile structures and MT speckles moved retrogradely (Fig. 1C; blue and red traces on the right side of the kymograph). In contrast, docked mitochondria and MT speckles moved anterogradely along the axon in a manner that appeared to be coordinated with the forward advance of the growth cone (Fig. 1C; green and red traces on the left side of the kymograph) (Video S1).

A challenge in analyzing the bulk motion of MTs is that it has a complex pattern that moves in space as growth cones advance. In previous studies, motion in growing neurons was analyzed by tracking individual objects in each frame by hand or by drawing lines across kymographs where the initial and final position of objects are determined to give a time-averaged velocity^11, 13^. The former has the advantage of providing a detailed description of motion even when it changes over time, but is time consuming and difficult to apply along the axon, where speckle density is high and motion is slow (i.e. at a rate less than 1 pixel per frame (p/f)). The latter method is more rapid but less precise because it uses just the initial and final position of a trace to calculate average motion. Both share the difficulty of aligning sub-cellular motion profiles in growing neurons because the internal reference frame moves relative to the substrate. Advances in optical flow and particle tracking routines now allow sub-cellular motion tracking with high temporal and spatial resolution with the caveats they are most reliable when the signal to noise ratio is high, the illumination conditions are steady, and the motion is slow^21, 22^. While we have previously used optical flow analysis to monitor the fast transport of brightly labeled mitochondria^23^, MT speckles are difficult to image and even under optimal conditions have relatively low signal-to-noise ratios^22^.

To address the issues of illumination and signal-to-noise, motion in kymographs was traced by hand using a drawing tablet (Fig. 1C), the traces were converted to movies, and optical flow analysis of the kymographs was conducted using code we developed in ImageJ called KymoFlow. In brief, it processes a kymograph with a novel optical flow algorithm and returns a kymograph where local motion is shown as a function of position and time. To address the issue of measuring fast motion, the program systematically rotates the time and distance axes of movies before processing. For example, when a kymograph is rotated by 90°, motion with a rate of 10 p/f is converted to a rate of 0.1 p/f. As a result, the strength of optical flow software in processing sub-pixel motion^24^ can be applied to rapid motion. Combining the rotated and unrotated output then provides estimates of fast and slow motion. To assess the accuracy of KymoFlow, we processed a set of 91 kymographs where a line was rotated from 0° to −90° in one degree increments. Because the angle of each line and hence the velocity is defined, the ‘ground truth’ flow is known^25^. To provide a reference for comparison, the same data set was processed with the Lucas Kanade motion tracking algorithm (LKMTA) implemented in the ImageJ FlowJ plugin^26^. Illustrations of the input and output for a subset of the angles are shown in Fig. 1D. For each angle, the mean and standard deviations of flow in units of p/f were measured for both algorithms. To avoid division by zero errors and infinite velocities, just the angles ranging from −1° to −89°, which correspond to 57.3 p/f and 0.0175 p/f were graphed (Fig. 1E). Examining the standard deviations indicates that KymoFlow produces more precise estimates of velocity compared to the LKMTA (Fig. 1E). In addition, the output of the LKMTA starts to deviate significantly from the expected velocity at 5 p/f as expected, while the KymoFlow produces accurate results up to velocities of 28 p/f. A second means to evaluate the accuracy of optical flow techniques, that circumvents issues with division by zero errors, is to calculate the average angular error between the output of an algorithm and ground truth flow^25^. In brief, the velocities which are in units of p/f are converted to angles in units of degrees, the mean angle for each image is measured, and this is subtracted from the ground truth angle, which is converted to the absolute value to calculate the absolute average angular error (AAE) in degrees (Fig. 1F). For the full set of angles, the AAE for the KymoFlow and Lucas Kanade programs were 0.4° and 4.2°, respectively, with the KymoFlow being significantly more accurate by t-test (p < 0.005). For angles ranging from −90° to −3°, which are most relevant to the analysis of experimental data, the errors were 0.22° and 2.3° (p < 0.0001). Since an AAE of less than 2° is typical of most state of the art motion tracking algorithms^27^, KymoFlow performs well in terms of the limited task of converting lines on a kymograph into maps of local motion.

Having developed and validated software for analyzing motion, traces of retrograde flow across the growth cone using DIC images and the motion of docked mitochondria and MT speckles (Fig. 1C) were processed to produce kymographs where the local pixel intensity was equal to the velocity of motion in units of μm/h. To spatially align reference points over time, the flow kymographs were sheared, so the position of the growth cone was constant over time (Fig. 1G). For each time lapse series, the average velocity of motion as a function of distance was then calculated (Fig. 1H) and aligned such that the point that MT velocity transitioned from rapid retrograde flow to slower anterograde motion was set at the zero point on the x-axis. Fig. 1I shows the averaged data sets from the 30 time lapse sequences where axons elongated. In the region of the growth cone that corresponds with the P-domain, MTs and filopodia/lamellipodia moved rearwards at average rates of −97 ± 21 μm/h and −151 ± 20 μm/h, respectively, while docked mitochondria and MTs along the axon moved at rates of 18 ± 6 and 13 ± 5 μm/h (mean ± 95% CI) respectively (Fig. 1I). Regression analysis revealed that the relative motion of MTs and docked mitochondria had an r-squared of 83% and slope of 0.83 ± 0.12 (mean ± 95% CI; p < 0.001 Fig. 1J). This suggests that while docked mitochondria track the motion of the underlying MTs, mitochondria move slightly faster, presumably through the action of motors that move them along MTs. Given the pattern of MT transport, a more precise criterion for defining a ‘docked’ mitochondrion may be that it moves at the same relative velocity as surrounding mitochondria. Collectively, these results indicate that MTs and docked mitochondria move anterogradely during axonal elongation in chick sensory neurons grown on laminin.

To analyze the contribution of rapid bidirectional transport of short MTs to the overall transport of MTs and axonal elongation^8, 10^, the velocities and number of MT speckles that rapidly passed the center point of kymographs were measured (Fig. 1C, red arrows). For the data set discussed in this section (n = 34 neurons), the average velocities in the anterograde and retrograde directions were 0.1 +/− 0.05 μm/sec and 0.09 +/− 0.08 μm/sec (ave.+/− sd, n = 337 and 255 speckles, respectively) and the flux of fast transported MTs in the anterograde direction was 0.5 MT/min and in the retrograde direction 0.38 MT/min. This yielded a net flux of 0.12 MT/min, thus on average 1 MT moved towards the growth cone every 8.3 minutes. This is similar to a previous report where a rapidly moving MT speckle was observed on average every 4.2 minutes and 87% of the time speckles moved in the anterograde direction^10^; which results in a net flux of 0.18 MT/min (Table 1). An estimation of net transported MT mass can be made by multiplying the flux by the average length of the MTs. Careful measurements have demonstrated that rapidly transported MTs have an average length in the range of 2.7 μm to 3.6 μm^8, 10^. Using the longer value for our data, the net flux delivers 26 μm/h of new MT to the distal axon. If on average there are 22 MTs per axonal cross-section^16^, this would be equivalent to 1.2 μm of axon growth per hour (Table 1). Naturally this estimate is sensitive to the estimates of MT length and number, nonetheless quantitative analysis of our data and previous studies (Table 1) suggests that rapid transport of MTs relative to the axonal framework makes a relatively small contribution to the net advance of MTs at least in the region of the axon studied here.

**Table 1:** Quantitative analysis of rapid MT transport. Data compare two prior studies, with our findings.

	(Wang and Brown)^10^	(He *et al*.)^8^	Our work
Anterograde flux (MT/min)	0.21	0.56	0.5
Retrograde flux (MT/min)	0.03	0.3	0.38
Net flux (MT/min)	0.18	0.26	0.12
Net flux (MT/h)	10.6	15.6	7.2
Average MT length (μm)	2.7	3.59	3.59
Transported MT length (μm/h)	29	56	26
Ave. # of MT per cross section	22	22	22
Net Axonal Growth (μm/h)	1.3	2.5	1.2

Anterograde flux and retrograde flux refer to the number of MTs that move past a point along the axon over a given period of time in the respective directions. Net flux is determined by subtracting retrograde flux from anterograde flux. The transported length of MTs per hour is calculated by multiplying net flux by the average length of the transported MTs. Since that parameter was not measured in our study, we used the larger of the two values^8^ from the previous studies. Rochlin *et al*.^16^ reports a value of 22 MT/axonal cross section in sympathetic neurons, which have a similar morphology to sensory neurons. Net axonal growth is calculated by dividing transported MT length over time by the average number of MTs per axonal cross section.

### Anterograde flow of axonal material during axonal elongation

**Figure 2:**
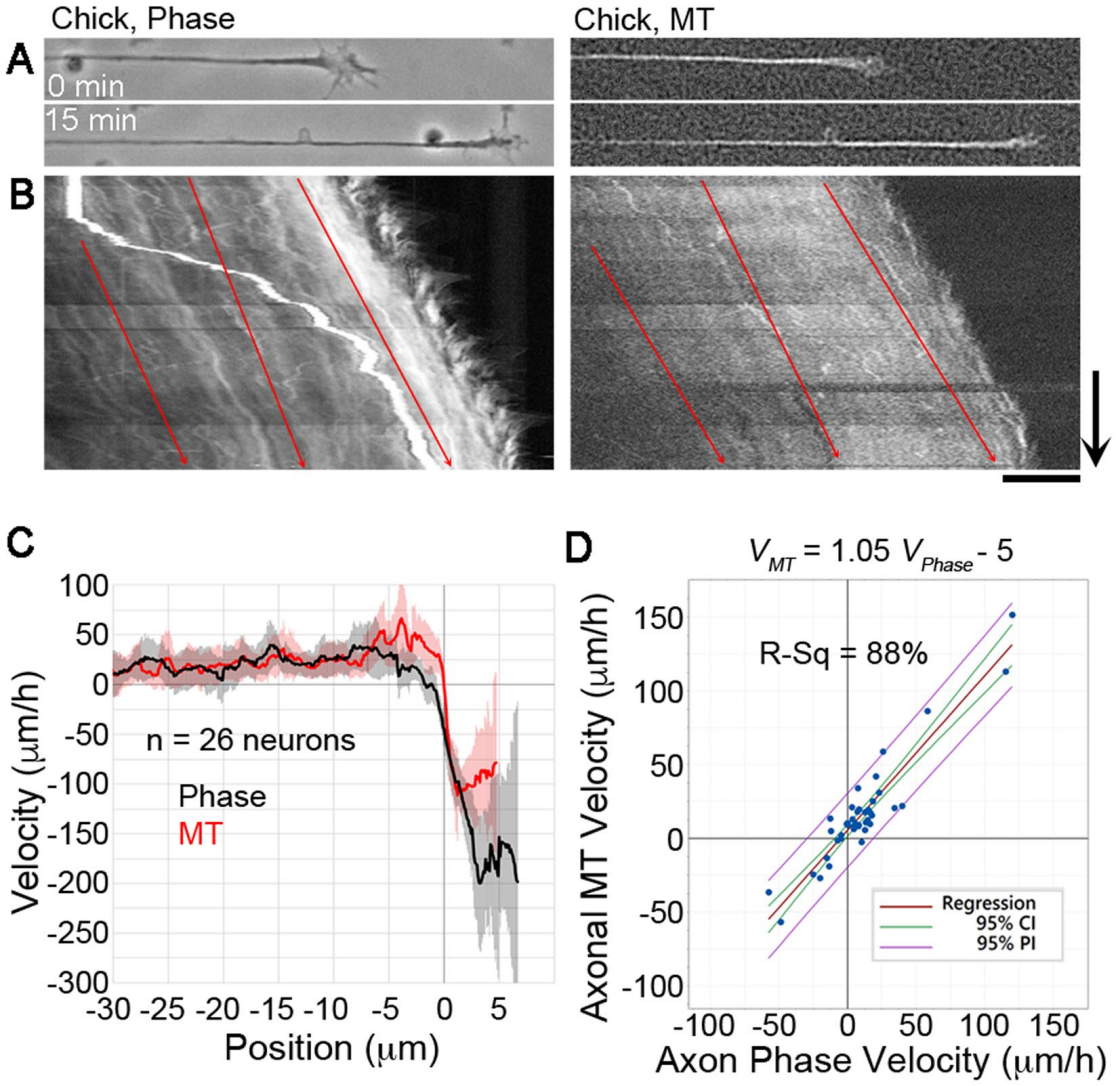
Phase-dense material and MTs move anterogradely during axonal elongation. (**A**) Phase contrast and MT FSM still images of a chick DRG neurite at the beginning and end of a time-lapse sequence. (**B**) Phase and MT kymographs showing anterograde flow; arrow = 5 min, bar = 10 μm. The red arrows are guides to illustrate motion. (**C**) Velocity ± 95% CI of phase-dense objects and MTs in 26 neurons. (**D**) Regression analysis of the motion of phase-dense objects and MTs.

The motion of MTs and mitochondria indicates that bulk forward translocation of materials in the axon makes an important contribution to axonal elongation. However, these are just two components. To address the more general question of whether axonal elongation occurs as the result of coherent motion of bulk axonal material, we acquired phase contrast images of chick DRG neurons, which reveals the distributions of axonal components based on their refractive index, while simultaneously monitoring MT translocation using FSM (Fig. 2) (Video S2). When processed into kymographs, phase imaging produces high contrast traces of motion along the axon and across the growth cone. In total, we examined 22 neurons for a combined time of 8:14 h. To correct for changes in growth cone velocity, these recordings were divided into 41 time-lapse sequences. Of these, 26 neurites elongated with an average velocity of 25 ± 10 μm/h (mean ± 95% CI) and 15 retracted at an average rate of −30 ± 20 μm/h (mean ± 95% CI). Tracing and analysis of the motion of MT speckles and phase-dense objects in kymographs was conducted as described above. For growing neurons, the average rate of motion of MTs along the axon was 26 ± 13 μm/h (mean ± 95% CI) and for phase-dense objects it was 21 ± 12 μm/h (mean ± 95% CI; Fig. 2C). These rates were not significantly different by two tailed *t*-test. In the P-domain of the growth cone, MTs moved retrogradely at an average rate of −100 ± 41 μm/h (mean ± 95% CI) and filopodia/lamellipodial veils moved at rate of −142 ± 34 μm/h (mean ± 95% CI). These were significantly different by two-tailed *t*-test with a p < 0.05. To assess the correlation between the motion of phase-dense objects and MTs, the average velocity of each along the axon were measured for each neuron and plotted (Fig. 2D). By linear regression the r-squared value was 88%, and the slope of the relationship was 1.05 ± 0.12 (mean ± 95% CI). These data suggest that the motion of MTs along the axon likely reflects the general bulk translocation of axonal material and is correlated with axonal elongation. Furthermore, these results suggest that phase contrast microscopy may be used to as a simple means to track bulk motion in neurons.

### MTs translocate anterogradely at the rate of neurite elongation

**Figure 3:**
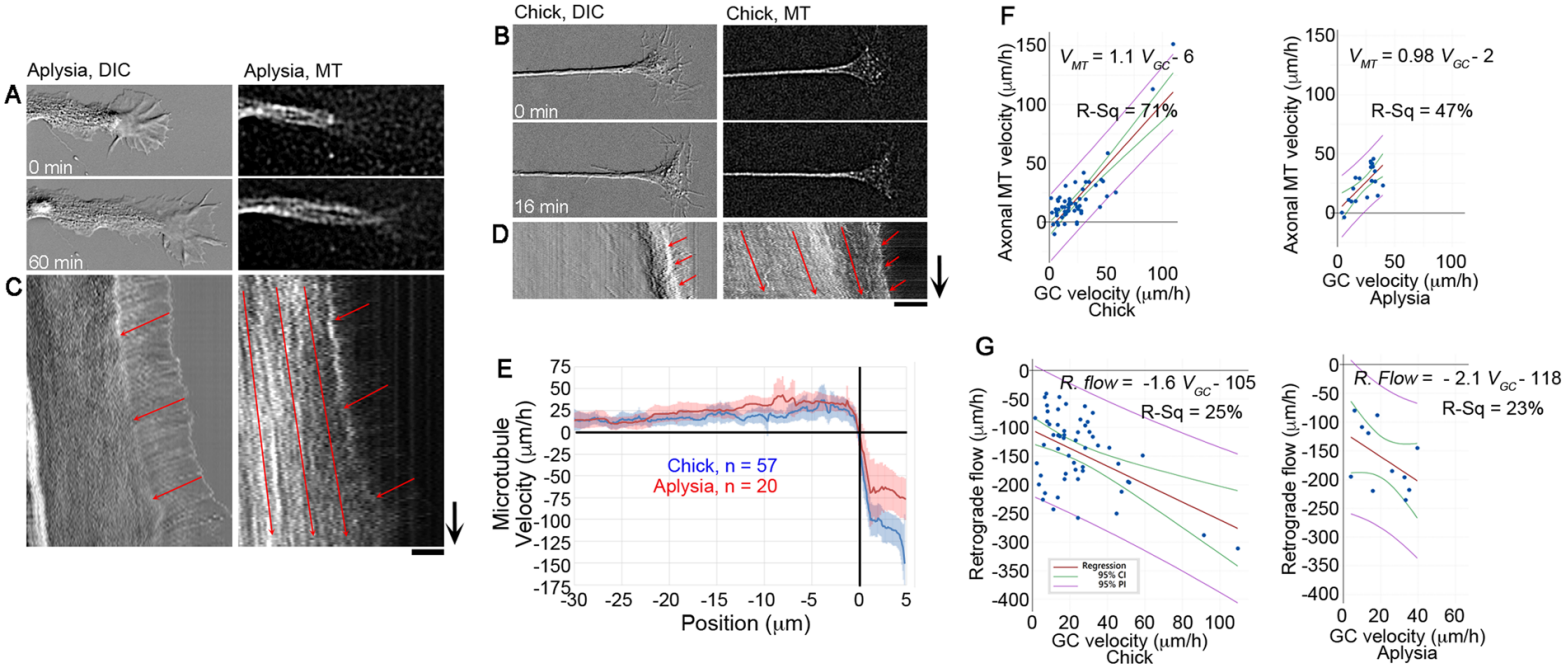
The pattern of MT motion is conserved between chick and *Aplysia*. Still images at the beginning and end of time-lapse sequences in (**A**) *Aplysia* and (**B**) chick neurons. Kymographs of (**C**) *Aplysia* and (**D**) chick neurons with red arrows indicating anterograde motion of MTs in axons and retrograde flow of phase dense material in the growth cone P domain; arrows = 10 min, bars = 10 μm. (**E**) MT velocity as a function of distance from the T zone. (**F**) Regression analysis of axonal MT and growth cone velocity. (**G**) Regression analysis of growth cone and retrograde flow velocity.

To better understand the relationship and degree of conservation between neurite elongation and MT advance, we used FSM and DIC imaging to track overall motion in *Aplysia* bag cell neurons and compared it to the analysis of MT motion in chick sensory neurons (Fig. 3A) (Videos S3 and S4). Many studies characterized the outgrowth of *Aplysia* neurons on poly-L-lysine (PLL) substrates, which causes growth cones to advance at slow average growth rates of about 2 μm/h^28^. Here, we examined patterns of MT movement in *Aplysia* neurites as they grew on the physiological substrate hemolymph, the circulatory fluid of *Aplysia* known to promote neurite outgrowth^29^. When compared with PLL substrates, neurites on hemolymph were thinner and more round-looking, and growth cones were generally smaller. Nonetheless, *Aplysia* growth cones generally maintained the well-known fan-shape and clear distinction of the central, transition, and peripheral domains typically observed on PLL substrates (Fig. 3A). In total, the growth of 20 *Aplysia* neurons was examined over a combined period of 22 h. On hemolymph, DIC time-lapse sequences collected simultaneously with MT FSM showed that on average, growth cones advanced at a rate of 21.6 ± 5.2 μm/h (mean ± 95% CI). To compare this with the outgrowth of chick sensory neurons (Fig. 3B), we pooled the data (n = 57) shown in Figs 1 and 2, which had an average rate of growth of 24 ± 5 μm/h (mean ± 95% CI). These neurite advance rates are in general agreement with previously published growth rates for both *Aplysia* neurons on hemolymph^29^ and chick neurons on laminin^14^, respectively, and are not significantly different by two-tailed *t*-test. To monitor MT translocation, FSM was used as shown in Fig. 1. Overall the velocity profiles of MT translocation in *Aplysia* and chick neurons were similar (Fig. 3E) with average rates of forward translocation along the axon of 18 ± 7 and 25 ± 7 μm/h for the chick and *Aplysia* neurons, respectively. Whereas the average rate was higher for the *Aplysia* neurons, the difference was not significant. To determine the relationship between the rate of anterograde MT translocation along the axon and velocity of growth cone advance, a regression analysis was conducted. In chick sensory neurons, we found that the correlation between growth cone and MT advance had an R^2^ value of 71% and slope of 1.1 ± 0.2. In *Aplysia* neurons, the R^2^ value was 47% and slope was 0.98 ± 0.5 (Fig. 3F). In both cases, the slopes were close to one indicating that on average there is a one to one relationship between the rates of anterograde MT translocation and growth cone advance. Nonetheless, the R^2^ values and the scatter of the data points indicate that elongation and MT advance do not occur in lock step, but are more likely to undergo cycles where growth cones advance more rapidly and slowly than MTs. Collectively these data indicate that the rate of MT translocation can account for the rate of growth cone advance, and that it is a conserved mechanism of growth in chick and *Aplysia* neurons.

Retrograde flow is a central aspect of growth cone motility. Nonetheless, the relationship between flow and growth cone advance, arising through natural variation has not been directly examined in neurons growing freely on physiological substrates. To investigate, we examined the correlation between the rate of growth cone advance and retrograde flow in individual neurons (Fig. 3G). In both systems, neurons that grow more rapidly tend to have higher rates of retrograde flow with slopes of −1.6 ± 0.8 and −2.1 ± 3 respectively (average slope ± 95% CI). By linear regression, the slope of the chick neurons was significantly different than zero with a p < 0.001, but for the *Aplysia* neurons significance was not reached p = 0.137. In both cases, this indicates that retrograde flow does not decrease significantly when neurons elongate more rapidly. The weak correlation with R^2^ values of 25% and 23%, respectively, suggests that factors in addition to the velocity of flow are likely to be important for modulating the rate of neurite elongation.

### MT assembly is required for bulk advance

**Figure 4:**
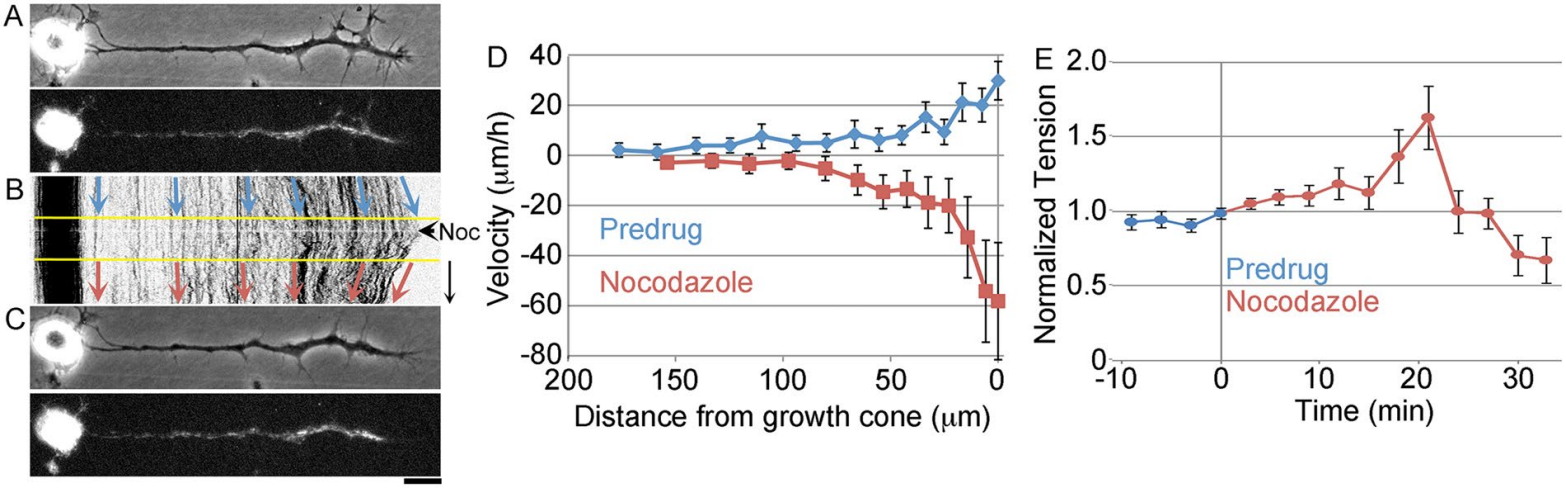
Disruption of MT assembly induces bulk retraction. (**A**) Still phase and fluorescent images showing the distribution of MitoTracker labeled mitochondria in a chick sensory neuron. (**B**) Kymograph illustrating the bulk forward advance of docked mitochondria before drug application, and bulk retraction following disruption of MT assembly with 1.6 μM nocodazole; arrow = 15 min. (**C**) Still images at the end of the time- lapse sequence; bar = 20 μm. (**D**) Velocity profile of the motion of docked mitochondria before and after drug treatment (n = 31 neurons). (**E**) Normalized neurite tension before and after drug treatment (n = 10 neurons).

A classic model for axonal elongation is that MT assembly in the growth cone lays down new MT tracks that promote and support the advance of the growth cone^7^. Our data suggests that bulk translocation quantitatively accounts for the majority of MT advance in the distal axon (Fig. 3F). This begs the question of “why is MT assembly required for axonal elongation”? To investigate, we tracked the motion of docked mitochondria in chick sensory neurons before and after the addition of nocodazole, a drug which suppresses MT dynamics (Fig. 4, video S5). We chose a concentration of 1.6 μM because it suppresses MT dynamics, without inducing complete disassembly of MT in biochemical studies and blocks axonal elongation without inducing axonal retraction during chronic bath application^16, 30^. Before the addition of nocodazole, axons elongated at a rate of 29 +/− 11 μm/h (average ± 95% CI, n = 31 neurons) and along the axon docked mitochondria advanced with a velocity profile that indicated relatively rapid bulk translocation in the region of the neurite directly adjacent to the growth cone and a declining rate of forward advance as distance from the growth cone increased. This profile of motion is similar to that seen in previous reports, and we have suggested that it arises because adhesions between the substrate and axon generate friction^13, 31^. After disruption of MT assembly with nocodazole, growth cones in phase images retracted at a rate of −27 +/− 24 μm/h (average ± 95% CI, n = 31 neurons) and docked mitochondria in the distal 75 μm of the axon retracted with a velocity profile that was roughly a mirror image of that seen in growing neurons. In addition, docked mitochondria further than 100 μm from the growth cone became stationary relative to the substrate (Fig. 4D). This suggests that acute disruption of MT assembly inhibits axonal elongation because it induces bulk retraction of material in the distal axon.

A logical explanation for the bulk retraction of material following disruption of MT assembly is that it increases axonal tension, which overcomes the net forces that are moving material forward in bulk. To test this, we attached the growth cones of chick sensory neurons to force calibrated needles, allowed them to attain steady force balance and then measured the tension by monitoring the deflection of the towing needle. Consistent with a previous study^32^, we observed a transient increase in tension which increased by approximately 60% from the baseline levels at 15 minutes after drug application (n = 10 neurons) (Fig. 4E). This suggests that disruption of MT assembly inhibits the bulk forward translocation of MTs by altering neuronal force balance.

## Discussion

Whether neurites elongate through the transport of tubulin that is then assembled into new MTs at the growth cone or because pre-assembled MTs flow forward has been an open question in cellular neurobiology for decades^7, 33^. Both MT assembly and translocation occur in elongating axons; however, the relative importance of these two processes as well as their functional relationship have remained unclear. The answer to this problem is important with respect to our basic understanding of the cellular mechanism of axonal elongation and in directing approaches for promoting axonal regeneration following injury or disease^34, 35^.

Here we report that neurite outgrowth is highly correlated with bulk MT translocation. Nonetheless, over the past several years a series of studies have reported the rapid bidirectional transport of short MTs through Stop-and-Go transport relative to a stationary array of long MTs^15, 36, 37^ and ‘MT sliding’ in *Drosophila* neurons^9, 38, 39^. An important question is how MT sliding and Stop-and-Go transport are defined and related. They are similar in that individual MTs move asynchronously in the anterograde and retrograde directions, at rapid rates characteristic of MT motors. Yet, they appear to differ from each other in several ways. MT sliding occurs in sub-cellular regions where MT polarity is mixed, such as in the neuronal cell body and at the tips of axons following severing^9, 40^. It does not occur along established axons where MTs are in parallel arrays, but rather is particularly important for the initiation of new neurites^9^ and the initial process of axonal regeneration after axonal severing^41^. It is driven by Kinesin-1 interactions between anti-parallel MTs^39^ and is suppressed by the mitotic Kinesin Pavarotti/MKLP1^38^. In contrast, Stop and Go transport occurs along the length of axons^10, 15, 42^. It powers the rapid bi-directional transport of short MTs (~0.1–3 μm) through an array of long parallel MTs, is driven by cortical actin associated dynein^8^ and suppressed by mitotic motors Kinesin-5^43^ and Kinesin-12^36, 37^. As it reflects the bona fide transport of short MTs through the axon it appears more closely related to slow axonal transport^44–46^ than MT sliding. Our work defines bulk translocation as being unidirectional, slow (i.e. 1–100 μm/hr as opposed to the velocity of MTs motors which is between 0.05–5 μm/sec), and includes the coherent translocation of MTs, organelles and phase dense material (Figs 1 and 2). The slower speed is likely caused by the fact that actin and MT-based motors are involved in the bulk flow of cross-linked MTs. Because it involves the coordinated forward motion of the distal axon and growth cone C-domain (Fig. 3), we view bulk transport as being more closely related to cell migration, as opposed to the transport or sliding of individual MTs.

While MT sliding, Stop-and-Go transport, and bulk transport are all documented, their relative quantitative contributions to neurite outgrowth have not been analyzed. Here, we did not see any evidence of obvious bi-directional MT sliding in either chick or *Aplysia* neurites as observed during neurogenesis in *Drosophila* ^9^. We think this is likely because we focused on a growth period where axons are well established and have been demonstrated to have highly parallel arrays of MTs^47^. Likewise, while we observed Stop and Go transport, it appears to account for roughly 1–2 μm/h of new axon (Table 1) based on our observations and calculations from two careful studies where it was directly investigated^8, 10^. In contrast, analyzing the rates of bulk MT transport and growth cone advance in *Aplysia* and chick sensory neurons, indicates that the slopes of the regression lines are 0.98 and 1.1, respectively. From a quantitative perspective, this suggests there is not a compelling need for the rapid delivery of MTs or MT assembly in the growth cone to explain the advance of new MTs at the tips of growing neurons. Nonetheless, it is important to note that we only examined the most distal region of the axon. Close to the cell body, there is every indication that the bulk MT array is stationary relative to the substrate (Fig. 4D)^13, 31^. As there must be a robust transport mechanism that quantitatively delivers tubulin and other components from the cell body^48^, it is possible that Stop-and-Go transport makes a larger contribution to MT transport in the proximal axon.

Understanding the relationship between neurite outgrowth and net MT assembly, MT dynamics, and MT motion is an important problem in neuronal cell biology. A widespread approach has been to use drugs to disrupt MT assembly and then to examine neurite outgrowth. In a foundational study, Bamburg *et al*., (1986) asked the important question of whether axonal elongation primarily requires MT assembly in the cell body or growth cone^7^. To answer it, nocodazole and other MT drugs were focally applied over a wide range of concentrations to each location, and rates of elongation were observed. Since they found outgrowth was dramatically more sensitive to drug application at the growth cone, they suggested MTs in the axon are stationary and assembly in the growth cone drives outgrowth. While the drug effects on elongation were straightforward, the interpretation in terms of MT motion was questioned^49^. In particular, when it was found that the low concentration of nocodazole (i.e. 0.32 μM) could cause a decrease in growth cone MT density^19^, this suggested that nocodazole blocked elongation by disassembling MT that were moving forward in the distal axon, as had been raised as a potential concern^49^. In parallel, the role of MT dynamics in neurite outgrowth were investigated by systematically varying nocodazole concentration from 160 nM to 1.6 μM and examining rates of axonal elongation and total MT mass^16^. Consistent with the idea that dynamic MTs are needed for elongation, as nocodazole concentration rose, elongation slowed. Surprisingly, a low concentration of nocodazole (i.e. 160 nM) had essentially no effect on production of new axon volume or total MT mass. Instead the axons were shorter and thicker. This suggested that mild disruption of MT dynamics left the processes for material addition to the axon intact, but disrupted growth cone advance by some other mechanism. Further evidence for the importance of normal MT dynamics came from experiments demonstrating that vinblastine halted the forward motion of growth cones, but still allowed for side to side growth cone movement^17^. More recently, a study has shown that reducing MTs dynamics by taxol or depletion of the +tip protein CLASP slows elongation^20^. This leads to the nuanced view that MT dynamics (as opposed to net MT assembly) are critical for axonal lengthening, but not necessarily growth cone motility or mass addition to the axon. Nonetheless, why MT dynamics are needed to lengthen the axon has remained puzzling.

To develop a better understanding of the relationship between MT dynamics and translocation, we treated chick sensory neurons with the MT inhibitor nocodazole, tracked the motion of docked mitochondria and measured neuronal force balance (Fig. 4, Videos S5 and S7). We observed that disruption of MT assembly increased neuronal tension and induced the bulk retraction of material in the distal region of the axon. Based on our prior studies that show a similar increase in tension and inhibition of bulk transport occurs when dynein is disrupted with the inhibitor ciliobrevin^50^, we speculate that this occurs in part because nocodazole disrupts the association of EB-1^51^ and cytoplasmic dynein with the plus ends of MTs. As a result, the pushing forces that dynein generates to power bulk translocation decrease^52^, tension along the axon increases^50^, and MTs in the growth cone are pulled rearwards (Fig. 4). In addition, the observed changes in forces may also occur through a decrease in the pushing forces associated with MT assembly^32, 53^, a disruption of cortical dynein or kinesin association with MTs^8, 40, 50^, or through the upregulation of NMII contractile force generation along the axon via the Rho signaling pathway^54, 55^. Presuming that lower concentrations of nocodazole or vinblastine^16, 17^ cause a smaller rise in tension, we suggest that disruption of MT dynamics slows axonal lengthening because it alters neuronal force balance, which slows growth cone advance. As this does not appear to disrupt the addition of new material to the axon, axons become short and thick during chronic disruption of MT dynamics^16^.

**Figure 5:**
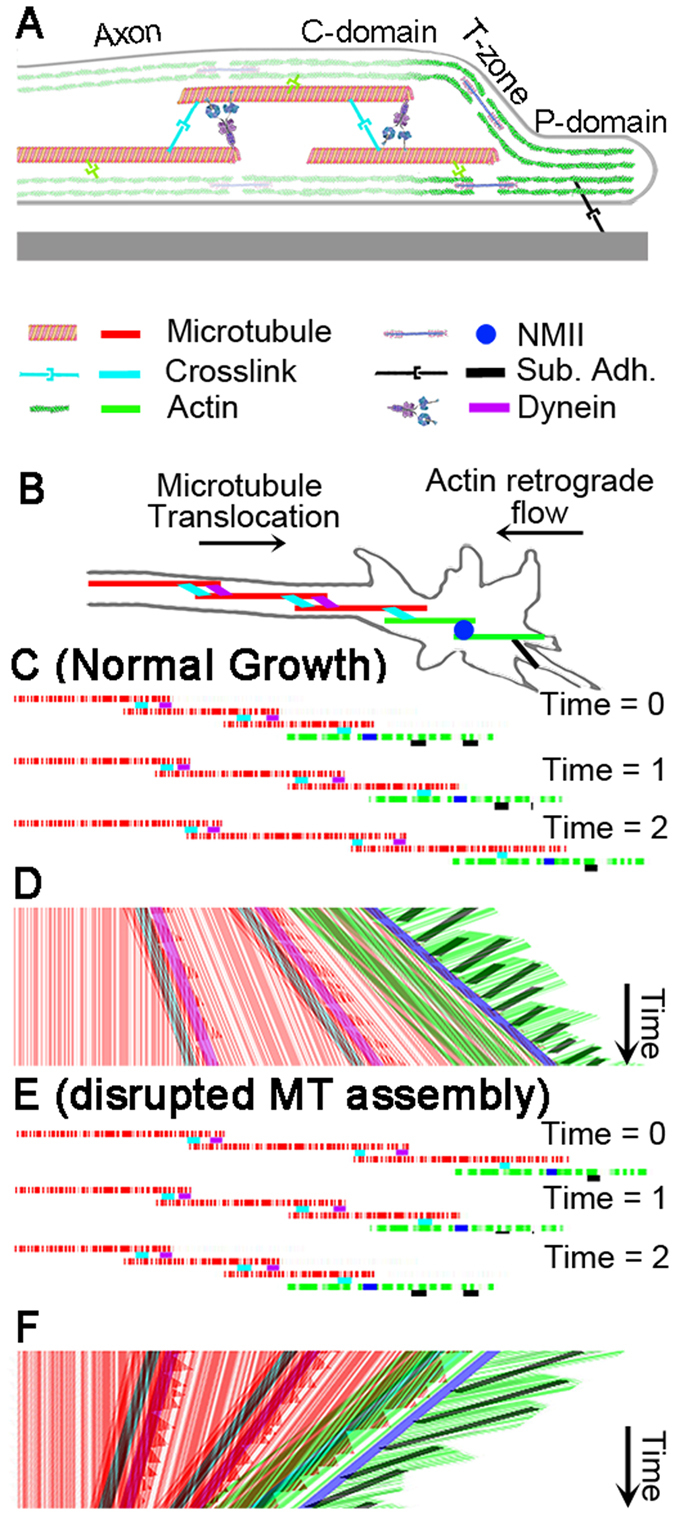
Model of neurite elongation by bulk forward translocation of MTs. (**A**) Side view of growth cone and distal neurite with important cytoskeletal elements. (**B**) Schematic of a growth cone and distal neurite with important cytoskeletal components and substrate adhesion (black). (**C**) Three MTs (red) and two actin filaments (green) labeled with fluorescent speckles at different time points. (**D**) Hypothetical kymograph of MTs (red), F-actin (green), motors (purple and blue), and cross linking proteins (light blue). Anterograde red lines reveal forward movement of MTs in the distal neurite and C-domain and retrograde green lines reveal retrograde movement of F-actin in P domain. (**E**) Illustration of MT and F-actin configuration under conditions where disruption of MT assembly induces retraction of the growth cone. (**F**) A hypothetical kymograph of MT and F-actin motion. While axonal frictional interactions with the substrate, as well as axonal actin and myosin activity are all important for both elongation and retraction, they are not shown to minimize the complexity of the figure.

Combining our current experimental results, which focus on movement patterns of MTs and refractile material in the growth cone and distal axon, with several previous studies leads to a speculative model for axonal elongation (Fig. 5) (Video S6). In this model, MTs in the distal axon move forward at approximately the rate of growth cone advance (Fig. 3F). Along the length of the axon, individual MTs slide apart through the action of motors such as dynein and kinesins^9, 36, 39, 50, 56, 57^ as well as forces generated in the growth cone that are transmitted along the axon^58–61^. Simultaneously, MTs undergo rounds of assembly and disassembly through dynamic instability, which over time leads to the addition of net MT mass^2, 14, 62^. When MTs polymerize or translocate into the P-domain, they are swept rearwards due to coupling to actin/myosin II-mediated retrograde flow^63, 64^. In terms of this hypothesis, the well-established substrate-cytoskeletal coupling model predicts that increased cell adhesion and clutching between cell adhesion receptors and the flowing actin cytoskeleton will result in reduced retrograde flow, rising traction forces and faster elongation because axonal MTs are pulled forward with a higher force^65–69^. In addition, we predict that increasing contractile force generation in the growth cone will also increase elongation by simultaneously increasing the forces that pull actin rearwards and axonal MTs forwards. This provides an explanation for the observation that myosin II inhibition decreases retrograde flow, traction forces, and axonal elongation when neurons are grown on endogenous substrates^67, 70, 71^, as well as the weak positive correlation between retrograde flow and elongation we observe (Fig. 3G). Finally, in terms of our model (Fig. 5E,F), MT assembly is required for axonal elongation because when it is disrupted neuronal force balance changes in manner that moves material rearwards in bulk (Fig. 4). This could occur because forces that push material forward decrease or because forces that oppose forward motion increase. Collectively, this provides a novel framework for understanding axonal elongation that could be further expanded to consider MT dynamics^2^, +tip proteins^72^, growth cone traction forces^73, 74^, the regulation of force generation by mechanosensing^58, 75^, non-linear clutch dynamics^76, 77^ and the effects of axonal force generation and friction on neurite outgrowth^61, 78, 79^.

By combining motion analysis of MT FSM, docked mitochondria, and phase-dense objects in two types of neurons from two species that are evolutionarily separated by roughly 600 million years^80^, we provide conclusive evidence that bulk movement of MTs and their attached cargo is highly conserved and correlated with neurite elongation. Collectively, the unique aspect of this study is that it is the first to provide a quantitative analysis that fully accounts for the addition of new MTs at the ends of growing axons. The observation that disruption of MTs alters neuronal force balance and blocks the bulk forward advance of material provides a straightforward explanation as to why dynamic MTs are required for neurite outgrowth. Yet how widely bulk MT translocation occurs during axonal elongation remains to be determined. At the moment, it is unknown if bulk MT translocation occurs *in vivo*. While we have visualized bulk translocation in embryonic *Drosophila* neurons *in vivo* ^81^, this was conducted using docked mitochondria. Given the historical and current importance of this problem^39, 82^, we think direct visualization of the motion of MTs *in vivo* is required to determine if our results reported here are relevant in living animals. In addition, these results do not exclude the possibility that axons elongate by net MT assembly or the transport of individual MTs under different growth conditions. In particular, when neurons are cultured on non-physiological substrates, experiments in *Drosophila* and *Xenopus* indicate bulk translocation is substantially reduced^81, 83^. Whether it persists to a limited extent^31^ or ceases and the addition of new MTs at the tip occurs by other mechanisms is unclear. Finally, whether the results of these studies conducted in PNS neurons apply to the growth of CNS neurons needs to be examined. As the cellular environment and intrinsic growth of CNS neurons is significantly different from PNS neurons, it is possible that different approaches to neurite outgrowth may have evolved.

## Materials and Methods

### Cell Culture

Chick dorsal root ganglion (DRG) sensory neurons were isolated as previous described^14^ from embryonic day 10–11 embryos obtained from the Michigan State University Poultry Farm and Purdue University Poultry Farm and grown in phenol red free L-15 media (Leibovitz’s L-15 medium, [ + ] L-Glutamine, Phenol Red supplemented with 0.6% glucose, 2 mM glutamine, 100 U/ml penicillin, 10% fetal bovine serum, and N9 growth supplement) on glass bottom dishes (35 mm petri dish, 10 mm microwell, No. 1.5 coverglass, MatTek Cooperation, Ashland, MA, USA) coated with 0.01% poly-ornithine solution for 1 hr at RT, rinsed 3x with sterile dH_2_O and then coated with 20 μg/ml laminin for 20 min. Unless otherwise noted, reagents were purchased from Sigma (St. Louis, MO, USA).

*Aplysia* bag cell neurons were cultured in L-15 medium (Invitrogen, Carlsbad, CA) supplemented with artificial seawater (L-15-ASW: L15 plus 400 mM NaCl, 10 mM CaCl_2_, 27 mM MgSO_4_, 28 mM MgCl_2_, 4 mM L-glutamine, 50 μg/ml gentamicin, 5 mM HEPES, pH 7.9) as described^84^. Glass-bottom dishes (World Precision Instruments, Sarasota, FL) were coated with 20 μg/ml poly-L-lysine (PLL; 70–150 kD) solution in hemolymph for 20 min at RT. Hemolymph was collected from adult *Aplysia* as previously described using a razor blade instead of a needle to pierce the animal^29^. Neurons were kept at RT until the time of injection; typically, 12 hours after plating.

Experiments involving chick embryos at Michigan State University were approved by the Michigan State University Institutional Animal Care and Use Committee by grant of an exemption based on the use of vertebrate non-mammalian embryos that are less than the half-way point of the incubation period. Experiments involving *Aplysia* and chick embryos at Purdue University were approved by the Purdue Animal Care and Use Committee (PACUC) by grant of an exemption based on the use of invertebrates (*Aplysia*) or chick embryos. All experiments were performed in accordance with relevant guidelines and regulations.

### Tubulin Microinjection

For chick neurons, microinjection pipettes (TW100F-4, World Precision Instruments, Inc. Sarasota, FL) were pulled on a Brown and Flaming horizontal pipette puller. For *Aplysia* neurons, microinjection pipettes (1B100F-4, World Precision Instruments) were pulled on a Narishige PP830 vertical pipette puller. Pipettes were then back-loaded with 1 mg/ml rhodamine tubulin (Cytoskeleton, Inc., Denver, CO, USA) in injection buffer (100 mM PIPES pH 7.0, 1 mM MgCl_2_, 1 mM EGTA) as previously described^84^. Before injection, tubulin was thawed, spun at 13,000 g for 30 min, kept on ice and back-loaded into pipettes pre-chilled to 4 °C. Microinjection was performed using the NP2 micromanipulator and FemtoJet microinjection system (Eppendorf North America, New York, NY), visualized with a Nikon ECLIPSE TE2000 microscope using phase contrast optics with a 40x objective. Alternately, microinjection was performed using a Narishige hydraulic micromanipulator with injection pressure supplied from a 3 ml luer-lock syringe.

### Mitochondrial Labeling

Mitochondria were labeled and imaged as described^14^ with 50 nM MitoTracker FM green (Invitrogen, Carlsbad, CA), incubated for 30 min, and allowed to recover in fresh L-15 for 2 h.

### Imaging

For simultaneous imaging of MTs and mitochondria, chick sensory neuron cultures were maintained at 37 °C, in 3 mL of phenol red free L-15 plus supplements covered with a layer of mineral oil, using a forced air heater and temperature probe on the stage of a Nikon ECLIPSE TE2000 microscope (Nikon, Inc., Melville, NY). Neurons were observed with a 60x Plan Apo VC/1.40 NA oil objective. Cells were illuminated with a 100 W Xenon lamp attenuated 98% with neutral density filters through a Texas Red cube (Chroma, Rockingham, VT) for visualization of Mito-Tracker. Transmitted light exposure for DIC was controlled with a VMM-D3 controller and CS25 shutter (Vincent Associates; Rochester, NY, USA). Fluorescent light exposure was controlled with a Lambda 10-C (Sutter Instruments). Micro-manager software was used to control the shutters and camera (Orca-ER digital camera CCD, model #CA742-95, Hamamatsu; Hamamatsu, Japan).

For simultaneous imaging of phase dense material and MTs, chick sensory neurons were imaged in regular supplemented L-15 media using a forced air heater controlled by a Variac Transformer, while evaporation was controlled by compensating water additions when the observations began. Time lapse sequences were recorded with a Photometrics CoolSnap HQ2 CCD camera on a Leica DMB microscope, with Lambda 10-C (Sutter Instruments) and Uniblitz Electronics shutters for the fluorescent and phase contrast channels, respectively, controlled by the ImageJ Micro Manager program (NIH). Observations were made with a 63x Plan Apo/1.32 oil phase 3 Leica objective, infinity/0.17/D.

Imaging of *Aplysia* neurons was performed with supplemented L15-ASW using a Nikon TE2000 E2 Eclipse inverted microscope equipped with a 60 × 1.4 oil immersion DIC objective and additional 1.5 magnification. Time-lapse sequences were recorded using a Cascade II charge-coupled device camera (Photometrics, Tucson, AZ) controlled by MetaMorph version 7.8.6 (Molecular Devices, Sunnyvale, CA).

### Nocodazole experiments

Chick sensory neurons were cultured on glass coverslips coated with laminin/poly-ornithine and mitochondria were labelled as described above. Images were acquired every 10 seconds for roughly 20 minutes, 1.6 μM nocodazole was bath applied, and then images were then collected for an additional 40 minutes. To analyse transport, kymographs were constructed by reslicing and z-projecting the movies in ImageJ. Rates of motion were measured for the first 15 min in the pre-drug condition and then for a 15 min period, starting 10 min after drug addition. Motion was measured by tracking the position of individual docked mitochondria over the time intervals as function of distance from the growth cone by drawing a line connecting their initial and final position and then recording the position and slope of the line using the analyse particle function in ImageJ. Data were exported to Excel for analysis. To enhance the contrast in the kymograph shown in the figure, the color was inverted, an unsharp mask function was applied and the contrast/brightness were adjusted. Measurement of neuronal tension using force calibrated towing needles was conducted as previously described^50^.

### Speckle processing and movement analysis

Images of fluorescent tubulin distribution were processed using a low pass filter and Laplacian transform in MetaMorph or with Gaussian blur and a Mexican hat filter in ImageJ. The processed sequences were then rotated, tightly cropped and converted into kymographs. To analyze the rapid motion of MT speckles, kymographs were opened in ImageJ, the line tool function was used to trace the position of a moving speckle over time, the analyze particle function was used to measure the slope of the line and data were then exported to Excel. Velocity was measured based on the slope of the line and flux was determined by counting the number of speckles that crossed the center point of a kymograph and then dividing this by the time period examined. For the analysis of bulk translocation, motion was traced by opening the kymographs in Photoshop and drawing lines over the paths of movement in separate layers with an Intuos Art tablet. The layers were saved as individual files which were batch processed in ImageJ with the KymoFlow software to produce kymographs where local pixel intensity corresponded to local flow. A description of KymoFlow is found in the supplemental data section. In cases were the velocity of elongation changed significantly, kymographs were divided into two or more sections. These were sheared with the ShearX plugin such that the position of the growth cone was aligned overtime, and the angle of the shearing was used to estimate growth cone velocity. To align velocity profiles, the position where MT translocation in the growth cone switched from forward to retrograde motion was used as a reference point. Data were then exported to Excel where the average velocity as function of position was measured and then averaged for each neuron along the axon and in the growth cone. Minitab was used for linear regression analysis. The source code and additional files needed for the KymoFlow program are available for download on GitHub at https://github.com/kmiller324/KymoFlow.

## Electronic supplementary material

Video S1. Axonal MTs and docked mitochondria translocate anterogradely during axonal elongation; Figure 1.

Video S2. Phase dense material and MTs move anterogradely during axonal elongation; Figure 2.

Video S3. Axonal MTs translocate anterogradely during axonal elongation of chick sensory neurons; Figure 3.

Video S4. Axonal MTs translocate anterogradely during axonal elongation of Aplysia Bag Cell neurons; Figure 3.

Video S5. Disruption of MT assembly induces bulk retraction of docked mitochondria in chick sensory neurons; Figure 4.

Video S6. Model of neurite elongation by bulk forward translocation of MTs; Figure 5.

Video S7. Model of neurite retraction by bulk forward translocation of MTs; Figure 5.

Supplementary Information
