# Supplementary material for: Neurite elongation is highly correlated with bulk forward translocation of microtubules
Source: Sci Rep. 2017 Aug 4;7:7292. doi: 10.1038/s41598-017-07402-6 (PMC5544698; doi:10.1038/s41598-017-07402-6)
Supplement: Supplementary file 8 — Supplementary Information [file 41598_2017_7402_MOESM8_ESM.pdf]

Supporting Information

Neurite elongation is highly correlated with bulk forward translocation of microtubules

Ahmad I. M. Athamneh, Yingpei He, Phillip Lamoureux, Lucas Fix, Daniel M. Suter and Kyle E. Miller

Supplemental Video Legends

Video S1. Axonal MTs and docked mitochondria translocate anterogradely during axonal elongation; Figure 1. Chick sensory neuron imaged using DIC and fluorescent microscopy to track mitochondria and microtubule speckles. As the growth cone advances, docked mitochondria and microtubules along the axon move forward, while filopodia and MTs in the P-domain move rearwards. The kymographs correspond to the movies above. Total time 16:50 min with images shown at 10 second intervals. Bar = 10 μm.

Video S2. Phase dense material and MTs move anterogradely during axonal elongation; Figure 2. Chick sensory neuron imaged using phase and fluorescent microscopy to track the motion of phase dense material and microtubules. As the growth cone advances phase dense material and MT speckles along the axon move forward while retrograde flow sweeps material rearwards in the leading edge of the growth cone. The kymographs correspond to the movies. Total time 14:54 min with images shown at 6 second intervals. Bar = 10 μm.

Video S3. Axonal MTs translocate anterogradely during axonal elongation of chick sensory neurons; Figure 3. Chick sensory neuron imaged using DIC and fluorescent microscopy to track microtubule speckles. As the growth cone advances microtubules along the axon move forward, while filopodia and MTs in the P-domain move rearwards. The kymographs correspond to the movies above. Total time 16:50 min with images shown at 10 second intervals. Bar = 10 μm.

Video S4. Axonal MTs translocate anterogradely during axonal elongation of *Aplysia* Bag Cell neurons; Figure 3. *Aplysia* Bag Cell neuron imaged using DIC and fluorescent microscopy to track microtubule speckles. As the growth cone advances microtubules along the axon move forward, while filopodia and MTs in the P-domain move rearwards. The kymographs correspond to the movies above. Total time 59:30 min with images shown at 30 second intervals. Bar = 10 μm.

Video S5. Disruption of MT assembly induces bulk retraction of docked mitochondria in chick sensory neurons; Figure 4. Chick sensory neuron imaged using phase and fluorescent microscopy to track docked mitochondrial motion and neurite outgrowth in response to disruption of MT assembly with 1.6 μM nocodazole. Prior to drug addition, the growth advances and docked mitochondria move forward in bulk. Following disruption, growth cone advance halts and docked mitochondria move rearwards in bulk. The kymograph corresponds to the movie above. Total time 59:40 min with images shown at 20 second intervals. Bar = 10 μm.

Video S6. Model of neurite elongation by bulk forward translocation of MTs; Figure 5. Movie shows speckle labeled microtubules in red and actin in green. Myosin II, shown in blue, generates a contractile force at the T-zone that drives retrograde flow. Substrate adhesions, shown in black, are pulled rearwards, which generates a traction force on the substrate. This is transmitted to microtubules in the C-domain through actin-MT cross-links. As a result, axonal microtubules are pulled forward and slide apart along the axon. MT dynamic instability occurs along the axon. Under conditions where net assembly occurs, MT length increases over time, which maintains the physical connectivity of the axon and prevents thinning.

Video S7. Model of neurite retraction by bulk forward translocation of MTs; Figure 5. Movie shows speckle labeled microtubules in red and actin in green. Under conditions where MT assembly is disrupted, MTs in the distal axon move rearwards in bulk. Based on experiments using force calibrated towing needles this is coupled with an increase in axonal tension. Based on the literature, this may arise through a decrease in pushing forces directly associated with MT assembly, disruption of pushing forces generated by dynein or the activation of myosin II contractile activity along the axon. The resulting change in neuronal force balance leads to bulk retraction of the distal axon and growth cone.

Supporting Methods

KymoFlow

KymoFlow takes a kymograph with high contrast lines and returns a kymograph where the local pixel intensity is equal to the slope of the line in units of pixels per frame (p/f). A central feature of KymoFlow is that it takes a kymograph, duplicates it and creates a series of kymographs which are rotated, processed and then averaged. The reason the kymographs are rotated is that the standard Lucas Kanade motion tracking algorithm (LKMTA) produces reasonable estimates of slow motion, but poor estimates of rapid motion, where many and in some cases most of the pixels generated have a null value (Fig. 1D). By rotating the input angles, the missing values for rapid motion can be filled in and pixels where the estimates do not converge can be eliminated. In more detail, a kymograph is first duplicated and the copy is rotated by 90º. The kymographs are then processed separately using the Lucas Kanade motion tracking algorithm [1] and then the 2nd kymograph is unrotated. The data in the resulting two kymographs are averaged, smoothed with a median filter and then all pixels that do not correspond to the input are set to not a number (NaN). This is then repeated eight times with the initial kymograph being rotated at 45º angles; in total each initial kymograph is processed 16 times. For some pixels, in particular at the beginning and ends of line, there is a large degree of divergence in the estimates; note the red dots at the ends of the traces in Fig. 1E for the LKMTA output. To identify them the standard deviation of the pixel intensity estimates is calculated and divided by the pixel intensity average. Pixels with a sd/ave > 0.2 are set to a null value. A strength of this approach is that the average absolute angular error is relatively constant for all input angles. Nonetheless, when objects undergo rapid motion the resulting horizontal line segments that appear in the kymographs are assigned high inaccurate velocities. As a practical means to suppress these errors, as a final step all pixels with an estimated motion greater than 50 p/f are set to 50 p/f. An obvious caution is that motion analysis is most accurate when it occurs at a rate of roughly 1 p/f in the kymographs; thus, it is best practice to acquire images or to adjust the time space ratio of the kymograph, so the motion of interest occurs at 1 p/f [2]. For our experiments, the pixel conversion factor is typically 1 p/f = 96 μm/h. Based on the data in Fig. 1F, which shows reasonable estimates can be made up to rates of 28 p/f, this indicates motion can be tracked with a reasonable degree of accuracy up to a rate of 2688 μm/h, which is roughly an order of magnitude greater than our motion of interest (Fig. 1I).

Analysis of flow error when using KymoFlow

To analyze the accuracy of optical flow algorithms, a set of test images were constructed in ImageJ by drawing a single line, 4 pixels thick and 250 pixels long that was rotated at 1º intervals from 0º to -90º without interpolation to create a set of 91 kymographs. These were then processed using the KymoFlow code or the Lucas Kanade motion tracking (LKMT) algorithm using a version of the FlowJ plugin in ImageJ that saves the x and y flow fields [2]. For the later, the kymographs were padded with 2 additional copies to make the line 3 pixels thick in z and 17 empty frames to make a stack of 21 images. This was resliced on the x-axis to make movies that were processed using the following settings: Gaussian deriv, Σ-s 2, Σ-t 2, τ 0.1, Σ-w 1 and Gaussian 1D. For a detailed description of these parameters see [2]. The x flow fields of each movie were opened as a stack, pixel values equal to 0.0 set to NaN, this was then resliced to create stacks of kymographs that were Z-projected using a plugin we developed called Z-project NaN.

Taking this output and that of KymoFlow for each angle, the mean and standard deviation of the pixel intensity histogram are measured to produce the output in units of p/f shown in Fig. 1D. To calculate the absolute angular error each kymograph was processed to convert the flow fields into units of degrees with the equation (angle = arctangent (velocity)*180/pi) and the histogram of each kymograph was generated to determine the estimate of the mean angle. The absolute value of the mean angle was subtracted from the input angle to yield the absolute angular error in units of degrees [3].

References

1 Lucas, B. D. & Kanade, T. in *IJCAI.* 674-679 (Morgan Kaufmann Publishers Inc., 1981).

2 Miller, K. E., Liu, X. A. & Puthanveettil, S. V. Automated measurement of fast mitochondrial transport in neurons. *Front Cell Neurosci* **9**, 435, doi:10.3389/fncel.2015.00435 (2015).

3 Barron, J. L., Fleet, D. J. & Beauchemin, S. S. Performance of Optical-Flow Techniques. *International Journal of Computer Vision* **12**, 43-77, doi:Doi 10.1007/Bf01420984 (1994).
